# Acetabular fractures in the elderly: modern challenges and the role of conservative management

**DOI:** 10.1007/s11845-021-02711-2

**Published:** 2021-07-19

**Authors:** Kunal Mohan, James M. Broderick, Hasnain Raza, Brendan O’Daly, Michael Leonard

**Affiliations:** National Centre for Pelvic & Acetabular Surgery, Department of Trauma & Orthopaedics, Tallaght University Hospital, Dublin, D24NROA Ireland

**Keywords:** Acetabular, Challenges, Conservative, Elderly, Fracture, Outcomes

## Abstract

**Introduction:**

The incidence of acetabular fractures in the elderly population is ever increasing. While management of acetabular fractures in young patients following high-energy trauma is well described, treatment of the elderly patient subgroup is complex and requires a unique, individualized approach. A variety of treatment strategies including operative and non-operative approaches exists to manage this vulnerable patient group. Conservative management of acetabular fractures in the elderly continues to play an important role in treatment of both stable fracture patterns and those medically unfit for surgery.

**Aim:**

This review assessing the current literature was undertaken with the purpose of summarising the challenges of management in this at-risk cohort as well as quantifying the role and outcomes following conservative management in the elderly.

**Conclusion:**

Our recommendation is that conservative management of acetabular fractures in the elderly can be considered as a treatment option on a case-by-case basis accounting for patient, injury, and surgical factors. If it is to be pursued, we advise a multidisciplinary approach focused on early mobility, minimisation of risk and regular follow-up to optimise patient outcomes.

## Introduction

The incidence of acetabular fractures in the elderly population is increasing [1]. While management of acetabular fractures in young patients following high-energy trauma is well described, treatment of the elderly patient subgroup is complex and requires a unique approach [2].

The objective of this article is to review the current literature in order to assess the challenges associated with management of these vulnerable patients and to quantify the potential role of conservative management in their treatment.

## Incidence

With advances in modern healthcare, the worldwide population is becoming increasingly elderly [3]. A corresponding rise in the incidence of acetabular fractures in the elderly of up to 23% per annum has been detected [4], frequently attributed to increasing levels of both longevity as well as activity within this subgroup [5]. Acetabular fractures represent up to 20% of all osteoporotic pelvic fractures [6] and are associated with significant patient morbidity [7]. An analogous increase in the incidence of these fractures in the elderly has also been described [5, 8], with a 2.4-fold increase in the proportion of acetabular fractures detected amongst the elderly population over a 27-year period [1]. This proliferation has resulted in acetabular fractures in the elderly representing the fastest-growing aspect of pelvic trauma [5], with further increases in incidence expected in the coming years [9], with an incidence of acetabular fractures of up to 32 per 100,000 predicted in over 75-year-olds [10].

## Challenges in the elderly

The objective of management of acetabular fractures is to optimise hip function in a method that allows for return to pre-injury levels of activity, minimising both the length of disability and overall complications [11]. While management strategies of acetabular fractures in younger populations are well described [12], management of these fractures in the elderly requires a unique approach, owing to the additional complexities conferred by both coexisting medical comorbidities and compromised bone quality typically encountered in this elderly patient group [2]. Advanced age has been described as a predictor for inferior outcomes following acetabular fractures [13]. A 1-year mortality rate of 8.1% following isolated acetabular fracture is described in those over 60 years of age across all treatment strategies, with the rate up to 25% in those presenting with concomitant injuries [14]. The potentially inferior outcomes in acetabular fractures in the elderly [15] have led to the need for specific treatment pathways to best approach these patients [16]. Elderly patients have been shown to have inferior outcomes than younger patients following injuries of all severities [17], with major injuries often underdiagnosed in the elderly population [18]. As such, robust initial diagnostic and treatment pathways allowing for prompt identification of injuries, relevant comorbidities and physiological vulnerability form a vital aspect of management of acetabular fractures in the elderly [19], with a particular focus on adequate resuscitation required in elderly patients suffering from acetabular fractures [20]. Additionally, a multidisciplinary approach has been suggested to be imperative in maximizing both functional outcomes and minimizing complications in this vulnerable patient group [21].

The challenges created by acetabular fractures in an elderly population are further evidenced by the types of fractures encountered. In contrast to the pattern usually seen in younger patients, acetabular fractures in the elderly mostly occur following low-energy injuries such as a fall from standing height [1]. Low-energy injuries of the acetabulum are typically associated with different fracture patterns than those encountered following high-energy injuries [22], with injuries often involving a direct fall onto the greater trochanter [23] and thus resulting in increasing involvement of the anterior column and quadrilateral plate when compared to younger patient groups [24].

**Fig. 1:**
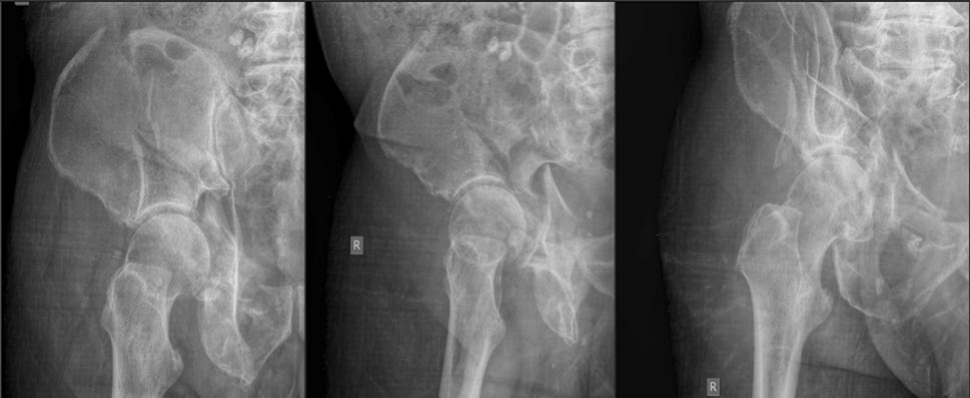
Index radiographs of typically encountered elderly comminuted acetabular fracture: index AP and Judet views

Potential underlying osteoarthritis or poor bone quality can lead to increased fracture comminution and displacement, resulting in atypical fracture patterns which make management increasingly challenging (Fig. 1) [23, 25]. A similar trend is also seen in posterior or both column acetabular fractures, which in the elderly are more likely associated with both marginal impaction and a posterior dislocation [1], each of which are predictors of inferior patient outcomes [26]. Given the likely complexity of fractures encountered, a diagnostic algorithm utilising plain radiographs, computerised tomography and 3D reconstruction is recommended to guide subsequent management [21, 27].

## Treatment strategies

The management of acetabular fractures in elderly patients thus presents a unique challenge to medical practitioners [2], with treatment strategies influenced by patient factors such as physiological and medical comorbidities, injury factors such as fracture pattern and associated injuries and treatment factors such as treatment timing and surgeon preference [28]. In the context of the absence of formal guidelines for treatment of these patients [29], treatment options are varied and require individualization based on the above factors [28].

Treatment is broadly subdivided into non-operative and operative options, with operative strategies including traditional open reduction and internal fixation (ORIF), minimally invasive stabilisation, total hip arthroplasty (THA) or a combination of approaches [30, 31]. Perioperative management of these injuries across all operative options is well-studied, with the need for timing of definitive surgery to factor in patients’ preoperative risk and early postoperative mobilisation described to maximise outcomes across all strategies [5, 19]. Surgical treatment of these injuries is well compared in recent literature [28, 30], with the role of THA in particular becoming increasingly prominent owing to its favourable reoperation and mortality rates when compared to ORIF [28]. The rise in popularity for surgical treatment in an elderly population to include THA has also conferred a number of benefits including the ability to allow early full weight bearing and being able to convey a painless, stable hip for the patient [32]. Nevertheless, such an approach is associated with an added technical complexity in this patient group owing to the often unstable nature of the underlying fracture potentially limiting implant positioning [32]. Additionally, it is relevant to highlight the potentially significant perioperative risk following acute THA in this patient cohort, with increased susceptibility to complications in what is often a physiologically fragile patient group particularly prominent in a combined THA/ORIF approach as regards blood loss and anaesthetic time [2, 31].

## The role of conservative treatment

Historically, conservative treatment of acetabular fractures has been proposed as a valid treatment strategy in the elderly [33]. Despite the prominence of a variety of surgical strategies for managing acetabular fractures [25, 28], conservative management continues to form part of current management algorithms in this group [30]. This is in direct contrast when compared to modern hip fracture management, wherein conservative management is seldom used despite affecting the same joint [34]. This inconsistency and the growing incidence of acetabular fractures in the elderly necessitates further understanding of when conservative management is suitable in this patient group [1, 5, 8].

A traditional indication for conservative management of acetabular fractures in the elderly is in either non- or minimally displaced stable fracture patterns [9] or those with intact articular acetabular surface and a congruent femoral head [35], with the reasoning behind this approach being poor underlying bone quality, a potentially low physiological reserve to endure extensive acetabular surgery and likely low functional demands in an elderly cohort affecting postoperative outcomes [36]. Additionally, both column fractures displaying secondary congruence between the acetabulum and femoral head have also been considered suitable for conservative management in this patient group [19, 21]. Lastly, conservative management can be considered for displaced acetabular fractures in the moribund patient in which underlying medical comorbidities preclude their ability to safely endure surgical treatment of their fracture [9, 15, 19, 36], or those who are non-ambulatory [21]. An acceptable rate of delayed conversion to THA following non-operative management of elderly comorbid patients managed non-operatively due to underlying medical conditions has also been shown, making this a potentially valid treatment option in those unsuitable for surgical treatment in the acute setting [37].

## Approaches to conservative treatment

**Fig. 2:**
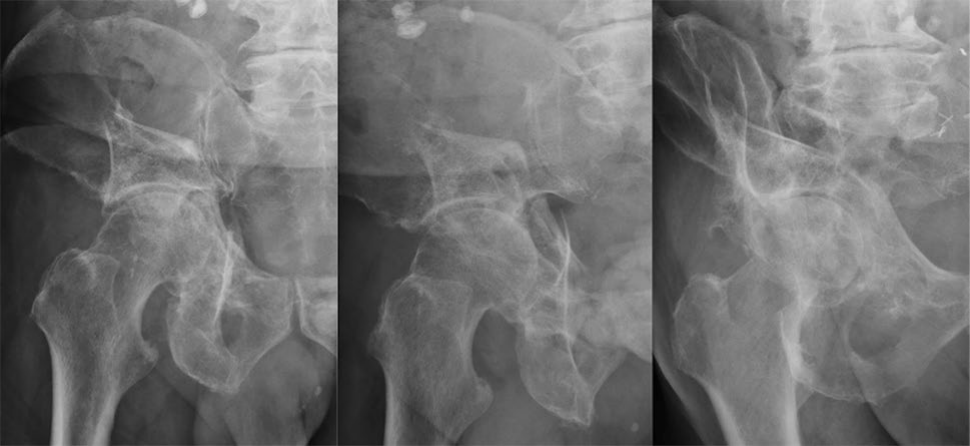
6-month follow-up radiographs following conservative management of elderly patient in Fig. 1: AP and Judet views

A structured approach to conservative management of acetabular fractures in the elderly is needed to maximize outcomes and reduce complications [19, 21, 36]. While periods of prolonged immobilization may potentially be advantageous in maintaining articular position [38], extended bedrest and traction, as what was historically performed, should not be undertaken due to both the unreliability of reduction and the associated complication rate described in traditional literature outlining this treatment regimen [39, 40]. Rather, a regimen of early mobilisation involving initial bed to chair transfer followed by transition to protected-weight bearing over the initial 6 weeks is suggested [24], so to avoid the sequelae of prolonged immobility such as pressure ulceration, respiratory deterioration, thrombosis and loss of function [21]. This should be supported by appropriate physiotherapy, pain management, thromboprophylaxis and osteoporosis workup where needed so to amplify patient outcomes [9]. Accompanying clinical and radiographic surveillance should be performed at regular intervals so to evaluate for both symptomatic improvement and fracture displacement (Fig. 2) [5], with the possibility for delayed surgical intervention to be considered in those with displacing fractures and associated pain limiting mobility and independence [36]. The importance of a systematic multidisciplinary approach to help maximise outcomes in the elderly patient with a conservatively managed acetabular fracture cannot be overstated, drawing upon modern principles of hip fracture management to help both minimise complications and maximise function in this patient group [41]. The multifaceted treatment strategy needed during conservative management of this at risk patient group relies upon a number of facets including clinical, radiological, functional, preventative and symptomatic measures [5, 9]. It is additionally imperative to appreciate that the conservative treatment strategy can be both time and resource-demanding, with a structured and regular multidisciplinary patient-centric approach to treatment as described for complex intraarticular fractures elsewhere in the body anticipated to confer better patient outcomes [42].

## Outcomes following conservative treatment

While traditional indications for conservative management of this injury in the elderly persist, functional outcomes following this treatment appear to be variable, despite the postulation that a bias to treat less complex fractures conservatively may also contribute to more favourable outcomes in this group [43]. While acceptable functional outcomes have been reported following displaced acetabular fractures in the elderly managed conservatively due to medical comorbidities [37], other studies have reported diminished outcome results with only 29% of patients returning to baseline ambulatory status following non-operative management [43]. When directly comparing functional outcomes following acetabular fractures in elderly patients, Boudissa et al. reported functional outcomes and post-injury autonomy status were significantly better in those managed surgically [44]. A possible contribution to this is that those deemed fit for surgical management may be a self-selecting group that were likely to do better notwithstanding their treatment due to their potentially favourable baseline.

Additionally, maintenance of articular reduction in conservatively managed acetabular fractures also appears to be reduced, with between 14.3 and 30% of patients being shown to maintain articular reduction following conservative management on follow-up radiographs [38, 45].

Overall outcomes following conservative management in the elderly appear to be limited, with an overall inpatient hospital length of stay (LOS) of between 11.1 and 20 days [45, 46] presenting potential risk to the patient. Post-operative independence is impaired, with only 23% of patients returning home and 19% mobilising independently following their injury [45]. A 1-year mortality rate of between 24 and 44% has been reported following conservative management [45, 46], with the mortality of conservatively managed acetabular or pelvic fractures demonstrated to be higher than the general population and closely resembling outcomes following neck of femur fracture [47].

## Recommendations from the National Centre for Pelvic and Acetabular Surgery

The incidence of acetabular fractures in the elderly population is increasing [1]. Management of this elderly subgroup of patients is increasingly complex, owing to both their underlying medical and physiological status, underlying bone quality and typically complex fracture patterns [2]. Outcomes following acetabular fractures in the elderly are typically inferior to those in the younger population [15], necessitating a systematic, multidisciplinary approach to all facets of the patient’s injury [16].

A variety of treatment strategies including operative and non-operative approaches exist to manage this vulnerable patient group [30], with an individualized approach required in treatment of these patients [28]. Unlike in modern hip fracture management [34], conservative management of acetabular fractures continues to play an important role in treatment of stable fracture patterns and those medically unfit for surgery [9, 15, 19, 21, 36]. Functional outcomes following conservative management are variable [37, 39, 40], with overall outcomes and survivorship limited when compared to the general elderly population [45–47].

As the national centre for management of pelvic and acetabular injuries, tertiary acetabular referrals of all ages are treated, with elderly patients making up a sizeable proportion of both non-operative and operative acetabular injuries encountered [48]. Our recommendations are that conservative management be considered as a treatment option on a case-by-case basis accounting for patient, injury and surgical factors. If conservative management is to be pursued, we further recommend a structured approach involving early mobilisation, multidisciplinary input and close follow-up to maximise success and minimize patient risk [5, 9, 21, 24, 36].
